# Size and site matter: the influence of corpus callosum subregional lesions on the magnitude of cross-education of strength

**DOI:** 10.3389/fphys.2025.1554742

**Published:** 2025-02-24

**Authors:** Marco Morrone, Gianluca Martinez, Antonio Achene, Mariano Scaglione, Salvatore Masala, Andrea Manca, Franca Deriu

**Affiliations:** ^1^ Department of Biomedical Sciences, University of Sassari, Sassari, Italy; ^2^ Department of Medicine, Surgery and Pharmacy, University of Sassari, Sassari, Italy; ^3^ Unit of Endocrinology, Nutritional and Metabolic Disorders, AOU Sassari, Sassari, Italy

**Keywords:** interlimb transfer, contralateral strength training, multiple sclerosis, MRI, rehabilitation, callosal damage

## Abstract

**Introduction:**

Cross-education is an established yet not fully understood phenomenon involving interhemispheric processes within the corpus callosum (CC) that result in strength gains in the untraining limb following training of the contralateral homologous muscles. There is a substantial lack of cross-education studies employing lesional models. This study employed the model of multiple sclerosis, a condition typically featuring demyelinating callosal lesions, to pinpoint CC subregions that mediate cross-education, potentially fostering the mechanistic understanding of the interlimb transfer.

**Methods:**

Nine individuals with relapsing-remitting multiple sclerosis (median Expanded Disability Status Scale: 3.5) and focal CC lesions underwent a 6-week, high-intensity isokinetic training program (≥80% maximal effort at 10°/s) targeting their stronger ankle dorsiflexors. Sagittal FLAIR MRI scans were segmented into five CC subregions (CC1–CC5), with lesion volumes quantified for each subregion. Strength (peak concentric torque at 10°/s) was measured bilaterally before (PRE) and after (POST) training to determine cross-education, defined as the percentage increase in torque of the untrained, weaker limb. Correlations between lesion volumes in CC subregions and cross-education were analyzed.

**Results:**

Both the trained (+21.5 ± 15.8%, p = 0.002) and untrained (+35.2 ± 24.9%, p = 0.003) limbs demonstrated post-training strength gains, reducing but not eliminating inter-limb asymmetry. Lesions specifically in the rostral body (CC2) correlated with reduced cross-education magnitude (rs = −0.670, p = 0.048) and smaller improvements in strength asymmetry (rs = 0.809, p = 0.008). No associations were detected in other CC subregions.

**Conclusion:**

These findings highlight the pivotal role of specific CC subregions, particularly the rostral body, in mediating cross-education of strength. These findings advance our understanding of CC role in the interhemispheric dynamics underpinning cross-education. Routine MRI can identify patients without CC2 lesions who may benefit from cross-education, providing a practical approach to improving muscle strength when weaker muscles cannot be directly trained.

**Clinical Trial Registration:**

ClinicaTrials.Gov, identifier NCT02010398

## Introduction

Cross-education is a long-known phenomenon first introduced in 1894 (Scripture et al., 1894). It refers to increased motor output (i.e., force generation, skill) of the opposite, untrained limb following a period of unilateral exercise training (Manca et al., 2021). This interlimb transfer of strength has been consistently observed in healthy individuals (Lee and Carroll, 2007; Coratella et al., 2022; Altheyab et al., 2024), orthopedic (Farthing et al., 2009) and neurological populations displaying predominantly unilateral motor impairment and muscle weakness, such as stroke survivors (Dragert and Zehr, 2013; Urbin et al., 2015) and persons with multiple sclerosis (Manca et al., 2017a; Manca et al., 2020) with reported strength gains in the untrained homologous muscles from aggregated data ranging from 9% to 18% in healthy subjects (Manca et al., 2017b) and up to 29% in mixed patient populations (Green and Gabriel, 2018a). The underlying neurophysiological mechanisms are thought to involve cortical and subcortical adaptations, including increased excitability and plasticity within motor networks of the untrained hemisphere. Interhemispheric interactions facilitated by transcallosal pathways are considered crucial for these adaptations (Green and Gabriel, 2018b; Lee and Carroll, 2007; Manca et al., 2018; Ruddy et al., 2017b).

The corpus callosum (CC) is the largest fiber bundle in the human brain, comprising approximately 300-million fibers that connect cortical regions across the two hemispheres (Hofer and Frahm, 2006). It plays a fundamental role in integrating sensory, motor, and cognitive information, enabling coordinated bilateral activities (Jokinen et al., 2007; Ruddy et al., 2017a; Wahl and Ziemann, 2008). Topographically, the CC is organized into subregions responsible for connecting specific cortical areas, each associated with distinct functional networks. While different segmentation schemes exist (Hofer and Frahm, 2006; Jokinen et al., 2007; Witelson, 1989), anatomically the anterior midbody (i.e., the section immediately behind the rostrum and the genu) connects premotor and supplementary motor areas (PMs, SMAs), whereas the posterior midbody (i.e., the midbody part ahead of the isthmus) links primary motor cortices (M1s) (Hofer and Frahm, 2006). These subregions facilitate the interhemispheric communication essential for motor ideation/planning, and execution (Fling et al., 2011). Despite compelling evidence indicating that transcallosal motor pathways play a key role in cross-education of skills following acute, single-session trainings (Ruddy et al., 2017b), to the best of our knowledge no studies have directly investigated the contribution of specific CC subregions to the magnitude of interlimb transfer of strength following chronic training. Understanding this structure-function relationship could be important for elucidating the precise neural mechanisms underlying cross-education and designing targeted rehabilitation protocols that harness interhemispheric signaling.

In this context, multiple sclerosis offers a unique opportunity to study how CC lesions influence cross-education of strength due to the impact of the disease on white matter integrity. Callosal pathology in multiple sclerosis is common and can disrupt interhemispheric communication, affecting motor coordination and cognitive functions (Degraeve et al., 2023; Hoppner et al., 1999; Kern et al., 2011; Peterson et al., 2017).

Clinically, individuals with multiple sclerosis frequently present unilateral weakness, spasticity, and impaired motor control (Larson et al., 2013), especially in the ankle dorsiflexion muscles (Lantis et al., 2024). Cross-education could serve as a valuable approach for these patients, allowing strength gains in the weaker muscles through training of the contralateral homologous stronger ones (Manca et al., 2017a). Since lesions vary in size and location within the CC, this population provides a “natural experiment” for exploring how specific patterns of callosal damage may facilitate or limit cross-education outcomes. In healthy cohorts, the integrity of interhemispheric pathways is generally preserved, thereby masking the specific contribution of distinct CC subregions to cross-education. By contrast, individuals with multiple sclerosis often exhibit isolated lesions in different segments of the CC, providing researchers with an opportunity to parse and differentiate how focal damage in regions linking homologous areas modulates the neurophysiological adaptation to unilateral training. New knowledge on this topic could have direct clinical relevance, as a better understanding of how CC integrity influences contralateral strength gains would help tailor rehabilitation programs to individual patients based on their neuroradiological profiles.

Based on the recognized role of transcallosal pathways in interlimb transfer and the signature callosal damages due to multiple sclerosis that could impair it, the present study aimed at identifying key callosal subregions involved in cross-education. We examined the effects of a 6-week, high-intensity unilateral training of the less-affected ankle dorsiflexors, starting from the hypothesis that the extent and site of lesions in key CC subregions would associate with the magnitude of strength gains in the contralateral, more-affected untrained limb.

## Methods

### Participants

Persons with multiple sclerosis were enrolled in the study from those referring to the Neurological Unit of the University Hospital of Sassari. Inclusion criteria were as follows: (1) definite diagnosis of MS; (2) clinical, radiological, and pharmacological stability for at least 6 months; (3) unilateral muscle weakness of the ankle dorsiflexors of at least 1 point on the Medical Research Council scale; (4) absence of white matter demyelinating lesions in the spinal cord, and underneath premotor, cingulate, and motor areas. Following the medical assessment, performed by a neurologist with a 15-year experience in MS evaluation and management, nine patients (7 females, 2 males) met all inclusion criteria and were included in the study.

### MRI segmentation and analysis of the corpus callosum subregions and their lesions

All MRI scans were acquired using a standardized protocol on a 1.5 T Philips Achieva® scanner (Best, Netherlands). Sagittal FLAIR sequences were utilized with the following parameters: repetition time (TR) = 4,800 ms, echo time (TE) = 297 ms, slice thickness = 1.5 mm with continuous axial slices, a matrix size of 200 × 200, and a field of view (FOV) of 240 × 240 mm^2^. Three neuroradiologists with over 20 years of experience in MRI analysis in persons with multiple sclerosis performed the MRI analysis and segmentation. Experts were blinded to clinical information. In cases where their initial segmentations or lesion detections differed, they reached a consensus through discussion.

Segmentation of the CC followed the approach described by Jokinen et al. (2007), dividing the corpus callosum into five subregions: the genu (CC1), rostral body (CC2), midbody (CC3), isthmus (CC4), and splenium (CC5). Lesions within the CC were defined as hyperintensities present across at least three consecutive slices. Manual segmentation of the CC was performed slice by slice on the sagittal planes, followed by verification in the axial and coronal views to correct any potential contouring inaccuracies or artifacts. After completing the manual segmentation, the open-source 3D Slicer software was employed to generate 3D reconstructions and conduct volumetric analysis.

The total volume of the CC was calculated and expressed in cubic centimeters (cc). Lesion volume within the CC was also measured in cc, and the lesion load was calculated as the percentage of the total CC volume occupied by lesions for each subregion. This approach provided a detailed analysis of both the structural integrity and the lesion burden within the CC in the study participants.

### Strength assessments

The maximal isokinetic peak torque of the ankle dorsiflexors on both the trained (less affected) and untrained (more affected) side was measured before (PRE) and after the intervention (POST) using isokinetic dynamometry (Biodex System-3, Biodex, Shirley, NY, United States). Participants were seated on the system’s chair with the trunk inclined at 85°, knee flexed at 30°, and ankle in full plantar flexion, stabilized with custom belts and straps. Participants underwent a light warm-up and familiarization with the system by completing two sets of 2–4 submaximal repetitions with a three-min rest in between at 10°/s isokinetic angular velocity. A short warm-up was chosen to avoid early onset of muscle fatigue, which can be pronounced in individuals with multiple sclerosis (Lambert et al., 2001) yet giving them enough practice to get acquainted with the isokinetic movement over the predefined range of motion. Following the warm-up, participants were instructed to dorsiflex as hard and fast as possible against the ankle attachment. Participants were asked to perform three maximal concentric contractions from full plantarflexion to their maximal dorsiflexion at an angular velocity of 10°/s for both sides. Peak torque of the dorsiflexors was recorded. The choice of testing and training concentrically was justified by the fact that patients had difficulties in actively dorsiflexing their ankle, particularly at the transition from push-off to swing where a concentric, open-kinetic chain contraction of the dorsiflexors is needed to ensure the clearance of the foot. A very low angular velocity (10°/s) was chosen to provide participants adequate time to achieve maximal torque, and to align with clinical isokinetic testing in persons with multiple sclerosis (Dvir, 2025). This facilitates higher torque outputs compared to faster velocities and takes into account the reduced neural drive and neuromuscular recruitment, which are signature features of multiple sclerosis (Kent-Braun et al., 1998). Dynamic isokinetic rather than isometric testing was chosen as it is more closely related to ankle function during gait, and to study participants’ capability to generate torque over the entire range of motion (Dvir, 2025).

Strength asymmetry was calculated both PRE and POST intervention as the difference between the trained and untrained sides using the following formula:

$$\text{Torque asymmetry}=\frac{\text{peak}\;\text{torque}\;\left(\text{trained}\right)‐\text{peak}\;\text{torque}\;\left(\text{untrained}\right)}{\text{peak}\;\text{torque}\;\left(\text{untrained}\right)}\times 100$$

Additionally, the gain in strength on the trained side and the cross-education effect on the untrained side were quantified in both Newton-meters (Nm) and percentage (%). During testing, visual feedback and strong verbal encouragement were provided to ensure maximal effort.

### Intervention

The intervention was administered using the same isokinetic device employed for the strength assessments. As per recent recommendations on transparent reporting of exercise variables in resistance training protocols (Coratella, 2022), the protocol consisted of high-intensity isokinetic concentric training of the ankle dorsiflexors of the stronger side. Participants completed three sets of four maximal efforts at 10°/s, with a three-min passive rest between sets. The ROM used was individualized for each participant according to baseline testing measurements. We chose to exercise the ankle dorsiflexors over a full ROM Before starting, participants underwent the same warm-up procedures described for the muscle strength measurements. The training was performed 3 days per week on alternate days over a 6-week period, with each session lasting approximately 30 min. All training sessions were supervised by a physiotherapist with a 10-year expertise in the rehabilitative management of persons with multiple sclerosis. As with the strength testing procedures, visual feedback and strong verbal encouragement were provided to maximize effort throughout the intervention.

### Statistical analysis

Volumetric data from the CC segmentations were obtained using 3D Slicer version 5.6.2. All statistical analyses were performed using SPSS version 29 (IBM Corp., Armonk, NY, United States). Descriptive statistics were used to report demographic and clinical characteristics of the participants, with continuous variables expressed as mean ± standard deviation (SD) or median and interquartile range (IQR) depending on the distribution of the data.

Normality and sphericity of the data were assessed using the Shapiro-Wilk and Mauchly’s tests, respectively. To evaluate the presence of strength asymmetry and cross-education effects, a repeated-measures analysis of variance (RM-ANOVA) was to test for changes in isokinetic peak torque of both the trained and untrained following the intervention. Main effects of TIME (PRE vs*.* POST), SIDE (trained vs*.* untrained) as well as TIME*SIDE interaction were tested. In case of significant differences, pairwise comparisons were carried out to locate the source of the difference. Effect sizes (Cohen’s d and partial eta-squared, η_p_^2^) were calculated alongside p-values to determine the magnitude of the observed differences. Effect sizes were interpreted taking (1) a Cohen’s d of 0.2 as small, 0.5 medium and 0.8 large; (2) a η_p_^2^ of 0.01 as small, 0.06 medium and 0.14 large (Cohen, 1988; Richardson, 2011). A p-value <0.05 was considered statistically significant for all analyses.

Correlation analyses were conducted using Spearman’s rank-order correlation to assess the relationship between lesion volume in the CC subregions and both the magnitude of cross-education and changes in strength asymmetry (Δstrength asymmetry). Correlation coefficients (r_s_) and p-values were reported to indicate the strength and significance of these associations.

## Results

### Participants’ characteristics

Nine persons with multiple sclerosis (7F, 2 M), aged 38 ± 13 years, withrelapsing-remitting course of disease satisfied the inclusion criteria and were included in the study. Demographic and clinical characteristics are detailed in Table 1 both at the individual and group level. Median EDSS was 3.5 (IQR: 2) indicating mild to moderate disability, with a mean disease duration of 11 ± 6 years. Four patients were on IFN-B1a, 4 on fingolimod and 1 on glatiramer acetate.

**TABLE 1:** Demographic and clinical characteristics of the participants.

	Subj. N1	Subj. N2	Subj. N3	Subj. N4	Subj. N5	Subj. N6	Subj. N7	Subj. N8	Subj. N9	Subjs 1–9^a^
Age (yrs)	48	43	32	27	33	52	24	59	20	38 ± 13
Sex	F	F	F	M	F	F	F	F	M	7F; 2M
Disease course	RR	RR	RR	RR	RR	RR	RR	RR	RR	9RR
Disease duration (yrs)	10	10	11	12	8	13	6	23	3	11 ± 6
EDSS	3.0	4.0	4.5	2.0	5.5	5.0	2.0	3.5	2.5	3.5 (2)^b^
DMT	IFN-β1a	IFN-β1a	Fingolimod	fingolimod	fingolimod	GA	fingolimod	IFN-β1a	IFN-β1a	

Abbreviations: yrs, years; RR, relapsing-remitting; EDSS, expanded disability status scale; DMT, disease modifying therapy; F, female; M, male; IFN, interferon; GA, glatiramer acetate.

^a^ All data reported as mean ± standard deviation.

^b^ EDSS reported as median (interquartile range).

### Corpus callosum lesions and segmentations volumetrics

**FIGURE 1:**
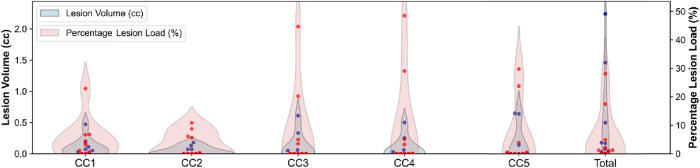
Distribution of lesion load and volume across subregions. Red dots indicate individual subjects’ lesion load, while blue dots represent individual subjects’ lesion volume.

Volumetrics analysis showed a mean total CC volume of 0.523 ± 0.791 cc. Lesion volumes in the five CC subregions are detailed in Table 2, while Figure 1 displays the distribution of lesions load and volumes across CC subregions.

**TABLE 2:** Corpus callosum lesion volumes by subregion and total.

Site of lesions	Subj. N1	Subj. N2	Subj. N3	Subj. N4	Subj. N5	Subj. N6	Subj. N7	Subj. N8	Subj. N9	Subjs 1–9^a^
CC1 (*cc*)	0.068	0	0.046	0.471	0.109	0.011	0.026	0.184	0	0.102 ± 0.151
CC2 (*cc*)	0	0	0	0.005	0.134	0.066	0	0.065	0.179	0.050 ± 0.067
CC3 (*cc*)	0	0	0	0.612	0.328	0.061	0	0.046	0	0.116 ± 0.214
CC4 (*cc*)	0	0	0	0.499	0.249	0.027	0	0.060	0.003	0.093 ± 0.172
CC5 (*cc*)	0.013	0.014	0	0.654	0.639	0	0	0.140	0	0.162 ± 0.278
Total (*cc*)	0.081	0.014	0.046	2.241	1.459	0.165	0.026	0.495	0.182	0.523 ± 0.791

Abbreviations: cc, cubic centimeters; CC1, rostrum and genu, CC2, rostral body, CC3, midbody; CC4, isthmus; CC5 splenium.

^a^ All data reported as mean ± standard deviation.

### Cross-education

RM-ANOVA on peak isokinetic torque showed a main effect of TIME (*p* = 0.001, η_p_^2^ = 0.744, *d* = 0.987) and SIDE (*p* = 0.005, η_p_^2^ = 0.656, *d* = 0.926), but no TIME*SIDE interaction (*p* = 0.076, η_p_^2^ = 0.659, *d* = 0.111). Pairwise comparisons showed a substantial asymmetry in isokinetic peak torque at baseline (29.3% ± 77.8%, *p* = 0.005, η_p_^2^ = 0.649, *d* = 0.919), with the trained ankle dorsiflexors generating 26.9 ± 9.2 Nm, and the untrained ones 20.8 ± 10.3 Nm. Post-training, strength asymmetry was still detected (*p* = 0.01, η_p_^2^ = 0.587, *d* = 0.838) yet reduced to 19.3% ± 59.9%. Subjects N5 and N9 were the only ones with a higher asymmetry POST (12.6% vs. 1.3%, and 9.2% vs. 1.6%, respectively).

Following the 6-week training there was an increase in strength of the trained side (+21.5 ± 15.8%, *p* = 0.002, η_p_^2^ = 0.718, *d* = 0.976) except for subjects N7 and N8, and in the untrained side (cross-education) (35.2% ± 24.9%, *p* = 0.003, η_p_^2^ = 0.689, *d* = 0.956), with no relevant differences by side. Individual and group-level data for the strength tests are reported in Table 3.

**TABLE 3:** Isokinetic strength test results.

		Subj. N1	Subj. N2	Subj. N3	Subj. N4	Subj. N5	Subj. N6	Subj. N7	Subj. N8	Subj. N9	Subjs 1–9^a^
PRE training	PT at 10°/s untrained side (Nm)	17	19.1	6.2	27.1	15.7	20	22.9	15.4	43.6	20.8 ± 10.3
	PT at 10°/s trained side (Nm)	22.4	26.0	20.5	39.5	15.9	26.0	26.2	21.0	44.3	26.9 ± 9.2
	Strength asymmetry (%)	31.8	36.1	230.6	45.8	1.3	30	14.4	36.4	1.6	29.3 ± 77.8
POST training	PT at 10°/s untrained side (Nm)	29.1	32	9.9	37.7	19.1	24.2	27.1	17.6	45.7	26.9 ± 10.9
	PT at 10°/s trained side (Nm)	31.7	37.2	26.1	42.1	21.5	30.9	27.1	22.1	49.9	32.1 ± 9.5
	Strength asymmetry (%)	8.9	16.2	107.1	11.7	12.6	27.7	0	22.6	9.2	19.3 ± 59.9
	Strength gain in trained side (%)	41.5	43.1	27.3	6.6	35.2	18.8	3.4	5.2	12.6	21.5 ± 15.8
	Cross-education effect in the untrained side (%)	71.1	67.5	59.7	39.1	21.7	21	18.3	14.3	4.8	35.3 ± 24.9

Abbreviations: PT, peak torque; Nm, Newton-metres.

^a^ All data reported as mean ± st. dev.

### Correlation analysis

**FIGURE 2:**
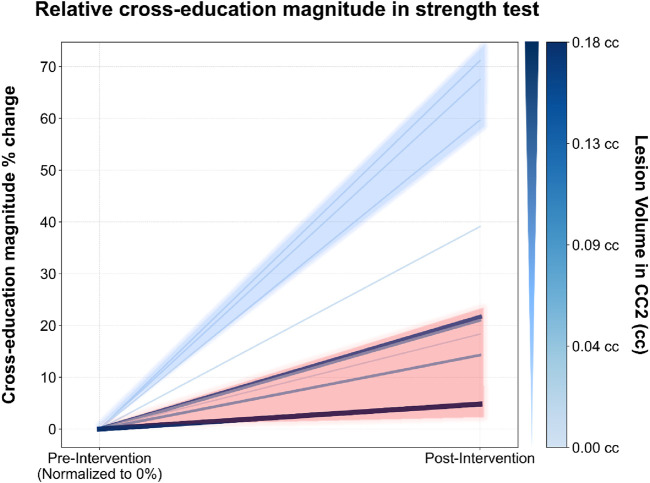
Relative cross-education magnitude (%) change in strength test before and after training. Each line represents an individual subject. The color and thickness of the lines indicate lesion volume in CC2 (rostral body) of the corpus callosum: thicker and darker lines correspond to larger lesion volumes. Cross-education magnitude is shown on the y-axis, with pre- and post-intervention time points on the x-axis.

Spearman’s rank-order correlation analysis revealed an association between lesion volume in CC2 and both cross-education magnitude (r_s_ = −0.670; *p* = 0.048), and Δstrength asymmetry (r_s_ = 0.809; *p* = 0.008). Conversely, no associations were observed between either variable and lesion volumes in the other CC subregions or with total lesion volume. Figure 2 illustrates the spaghetti plot depicting the individual cross-education magnitude and its correlation with lesion volume in CC2.

## Discussion

The present study was planned and executed to elucidate the relationship between callosal lesions due to MS and the magnitude of contralateral strength gains in the untrained limb (*i.e.*, cross-education). In doing so, we attempted to identify CC subregion/s mostly involved in the interhemispheric connectivity, a condition long known to lead to diffuse callosal damage and atrophy (Degraeve et al., 2023; Hoppner et al., 1999). CC, the major commissure interconnecting the two hemispheres, was chosen in view of its functional role that makes it the key anatomical node for the interhemispheric bidirectional interactions thought to mediate cross-education (Ruddy et al., 2017b).

The first finding of this interventional study was confirmation of cross-education occurrence in persons with multiple sclerosis (Manca et al., 2017a). The novelty resides in the fact that, despite evidence of diffuse callosal damage, the average magnitude of effect here observed overlapped with those observed in neurologically intact individuals (+15–30% strength in the contralateral homologous, untrained muscles) (Manca et al., 2017b). Following the 6-week strength training, the magnitude of cross-education in the untrained muscles was found to scale only with the lesional volume of CC2 subregion, which represents the portion of CC immediately posterior to rostrum and genu and anterior to midbody on the midsagittal plane (Jokinen et al., 2007). Importantly, if normalized by total CC volume, unlike the other subregions, even very low lesions in CC2 were associated with substantial reductions in the extent of cross-education, suggesting a major role of cortical areas interconnected at this level to ensure the interhemispheric inhibitory and facilitatory dynamics underpinning cross-education. Previous findings from seminal studies using diffusion tensor imaging (DTI) tractography (Huang et al., 2005) and functional magnetic resonance imaging (fMRI) activation in white matter (Fabri et al., 2011) support that CC subregions are associated with distinct functions of the human brain. According to this functional partition, anterior activation foci are associated with taste stimuli, central-anterior foci with motor tasks, central-posterior foci with tactile stimuli, and splenium foci with visual stimuli (Fabri et al., 2011). While the transcallosal connections of the cortical motor network have yet to be fully revealed, due to its complex architecture and the number and density of crossing fibers (Jeurissen et al., 2013), evidence from studies using resting-state functional connectivity of white-matter indicate that the anterior midbody is the most important interhemispheric hub for the cortical grey-matter sensorimotor system (Wang et al., 2020). Interhemispheric connections at this level bridge the two hemispheres (mainly PM and SMA) to be executed by the M1 to allow sharing of resources and information for the successful ideation and planning of voluntary movements.

Our finding on the influence of lesions specifically located in CC2 on the extent of interlimb transfer are founded on topographic studies that employed DTI-based tractography to evidence the massive crossing of fibers from PM and SMA to their contralateral homologous areas occurring in this specific subregion (Hofer and Frahm, 2006). More specifically, within the human CC, SMA-SMA crossing fibers are more in number and strength than those connecting M1 and dorsal premotor (PMd) cortices and pre-SMA (Fling et al., 2011).

Ruddy et al. (2017b) used a combination of neuroimaging methods (fMRI and diffusion weighted imaging-based tractography) to investigate the pathways in the human brain that mediate cross-education. They found elevated functional connectivity of the white matter pathways connecting bilateral SMAs in the resting motor network following a single session of ballistic unilateral, dominant-hand wrist flexion training that resulted in increased performance by >35% in the untrained wrist of healthy participants. However, the individual transfer did not correlate with the observed increases in activation. Conversely, in our cohort of persons with multiple sclerosis with callosal damage, we found robust association between the lesion load harming the integrity of interhemispheric fibers of the premotor system and the amount of individual transfer. The different duration, nature and site of the behavioral training administered in the two studies (6-week, ankle dorsiflexors’ high intensity resistance training in our case, single session wrist flexors’ ballistic training in Ruddy et al.‘s study) may be accounted for the discrepant neuroradiological-functional relationships observed. In a secondary analysis by the same group (Ruddy et al., 2017b), data on corticospinal excitability (MEP recruitment curves by single-pulse transcranial magnetic stimulation, TMS) were also appraised, revealing no neurophysiological correlates of the improved performance in the untrained limb. This was in line with a previous TMS study, which also assessed M1 intracortical facilitation, M1-M1 interhemispheric inhibition and sensory-motor integration and revealed no evidence of neural adaptations following 4 weeks of unilateral training of the intrinsic muscles of the hand (Manca et al., 2016). Overall, these reports seem to confirm that the acute adaptations that underpin the contralateral performance gains following unilateral training would be mediated by neural elements other than those occurring between M1 cortices or within M1, though the latter has been the typical target of TMS-based investigations attempting to elucidate the neurophysiological mechanisms of cross-education, with mixed results due to the paucity of studies that shared common hypotheses, outcomes and procedures (Hortobagyi et al., 2011; Manca et al., 2018).

Our findings align with an increasing corpus of studies that locate in the premotor rather than primary motor areas the anatomo-functional site of motor control of unilateral and bilateral movements and interhemispheric transfer, as well. However, while most investigations have dealt with acute exercise generally targeting hand muscles, we demonstrated a negligible contribution of M1 following a chronic (18 sessions) intervention targeting the lower limb, as demonstrated by the lack of association between CC3 (where most M1-M1 connecting fibers cross) and cross-education. Indeed, while CC3 was the subregion with the largest cumulative lesional load (along with CC5), lack of anatomical integrity did not translate in reduced magnitude of the transfer.

In a translational perspective, the present data bridging specific anatomical lesions to function may hold potentially relevant clinical implications. Beyond the observed increase in strength in the weaker, untrained side, the training here administered reduced the group-level asymmetry recorded at baseline, thus mitigating the common warning of clinical practitioners and neurorehabilitation specialists that contralateral training may enhance interhemispheric imbalance and strength/skill asymmetry. This consideration is in line with data showing no worsening of asymmetry with contralateral training of the stronger muscles (Manca et al., 2020), and with a recent consensus statement on cross-education where topic experts agreed that asymmetry is less important if there are benefits for the more affected limb (Manca et al., 2021).

### Study limitations

The first limitation to the findings of this study relates to the stringent criteria applied to identify candidate persons with multiple sclerosis who displayed callosal lesions but intact spinal cord. Additionally, to be enrolled they had to be free from evidence of lesions in motor and premotor areas. Consequently, the sample size was reduced to a cohort of highly selected persons with multiple sclerosis, narrowing the external validity and generalizability of our results to populations with similarly isolated neuroradiological features.

Another limitation is the lack of neurophysiological testing, which would have allowed to directly test, among others, hypotheses on PM-PM, SMA-SMA and M1-M1 connections and intracortical circuits, and their integrated influence on cross-education. Future studies paralleling neuroradiological methods with TMS-based protocols probing inhibitory and facilitatory intracortical (both ipsi- and contra-laterally to the training) and interhemispheric dynamics in the context of cross-education are warranted.

## Conclusion

Data showed that both site and size of the lesions influence cross-education. In particular, it was shown that the presence of lesions in the rostral body directly affected cross-education and that the lesion size was associated with the transfer magnitude. This new knowledge can be immediately translated into practice considering that it is based on a routine examination (conventional MRI), as patients who are not able to fully exercise their weaker side and do not display lesions in this subregion might be considered good candidates to cross-education protocols. Such information would allow the development of targeted rehabilitation strategies for persons with multiple sclerosis and other populations with callosal pathology, optimizing the use of cross-education to improve muscle strength.

## Patient consent statement

A written consent was obtained by all participants.

## Data Availability

The raw data supporting the conclusions of this article will be made available by the authors, without undue reservation.

## Author’s note

This article has received the Best Oral Presentation Award (presenter: Marco Morrone) at the 74th Congress of the Italian Society of Physiology (SIF).

## Abbreviations

CC, corpus callosum; M1, primary motor cortex; MRI, Magnetic resonance imaging; PM, premotor area; SMA, supplementary motor area; TMS, Transcranial magnetic stimulation.
